# Systemic antibiotic use in fire‐affected koalas ( *Phascolarctos cinereus* ) admitted to two wildlife treatment facilities during the 2019–2020 wildfires

**DOI:** 10.1111/avj.70006

**Published:** 2025-08-05

**Authors:** FK McDougall, O Funnell, JM McLelland, C Flanagan, M Govendir, F Stoeckeler, I Smith, ML Power

**Affiliations:** ^1^ School of Natural Sciences, Faculty of Science and Engineering Macquarie University Sydney New South Wales 2109 Australia; ^2^ Zoos South Australia Frome Rd Adelaide South Australia 5001 Australia; ^3^ Port Macquarie Koala Hospital, Koala Conservation Australia, Port Macquarie New South Wales 2444 Australia; ^4^ Sydney School of Veterinary Science The University of Sydney Sydney New South Wales 2006 Australia; ^5^ Kangaroo Island Veterinary Clinic Kingscote South Australia 5223 Australia

**Keywords:** antimicrobial resistance, antimicrobial stewardship, burns, bushfires, chlamydia infections

## Abstract

Antimicrobial resistance (AMR) is a global health threat for people and animals, including wildlife. The overuse and misuse of antimicrobials continues to fuel the spread of AMR. We performed a retrospective analysis of systemic antibiotic administration in fire‐affected koalas admitted to two wildlife treatment facilities during the catastrophic Australian wildfires in 2019–2020, to assess the practice of antimicrobial stewardship during a wildlife emergency. Triage and treatment records were obtained for fire‐affected koalas (*n* = 355) admitted to two facilities during the wildfires. Analyses showed that 40.3% of koalas at Facility A and 35.0% of koalas at Facility B received systemic antibiotics. The majority of antibiotics (63.9%, comprising multiple types of beta‐lactams) administered to koalas at Facility A were prophylactic treatments in koalas with noninfected moderate to severe cutaneous burn wounds. The majority of antibiotics (75.0%, comprising chloramphenicol and enrofloxacin) administered to koalas at Facility B were chlamydial disease treatments. Overall, 29.4% of all antibiotic treatments (predominantly beta‐lactams and enrofloxacin) were administered for recorded clinical infections. Where koala‐specific guidelines and protocols for treating burn wounds were not available, there was an overuse of systemic antibiotics and frequently sub‐optimal antibiotic stewardship in burnt koalas during the 2019–2020 wildfire emergency response. Best practice antibiotic prescribing was also not always feasible due to a shortage of first‐choice antibiotics (e.g., injectable chloramphenicol for chlamydial disease). This study highlights the importance of preparedness for future wildfire events and identifies a need for equipping veterinarians with guidelines for treating fire‐affected koalas during emergency situations. Additional education, guidance and resources are required to enable appropriate antimicrobial stewardship by responding veterinarians during wildlife emergencies.

**Abbreviations**
AMR: antimicrobial resistance
ASTAG: Australian Strategic and Technical Advisory Group on Antimicrobial Resistance
BID: twice per day
d: days
EM: empirical
h: hours
IM: intramuscular injection
IV: intravenous injection
kg: kilogram
mg: milligram
n: number
NSW: New South Wales
PCR: polymerase chain reaction
PK: pharmacokinetic
PO: per Os (oral administration)
q48h: every 48 hours
q72h: every 72 hours
q7d: every 7 days
q8h: every 8 hours
qPCR: quantitative PCR (real‐time PCR)
SA: South Australia
SC: subcutaneous injection
SCQ: subcutaneous injection
SEM: standard error of the mean
SID: once per day
VIC: Victoria

Antimicrobial resistance (AMR) is now recognised globally as a major threat to the health of animals (companion, livestock and wildlife).^1^ The spread of antimicrobial‐resistant bacteria threatens our ability to effectively treat infections in an array of species.^2^, ^3^, ^4^, ^5^, ^6^ The misuse of antimicrobials, including overuse, underuse and poor administration management, continues to fuel the emergence and spread of AMR.^1^ A key component of global AMR strategies,^7^ inclusive of Australia's National AMR Strategy,^8^ is to guide human health professionals and veterinarians to appropriately prescribe, dispense and administer antimicrobials.

Antimicrobial‐resistant pathogens circulate between people, animals and the environment; thus, a multisectoral or “One Health” approach is required to prevent and control AMR.^1^ As part of this One Health approach, the World Organisation for Animal Health has compiled a list of antimicrobials of veterinary importance^9^ that complements the World Health Organisation's list of Medically Important Antimicrobials,^10^ with the joint aim of preventing or minimising the spread of AMR between diverse pathogens. The Australian Strategic and Technical Advisory Group on Antimicrobial Resistance (ASTAG) has also developed Australian‐specific importance ratings for antimicrobials used to treat humans and animals in Australia.^11^

In the 2019–2020 Australian summer, unprecedented catastrophic wildfires occurred across Australia, burning almost 12.6 million hectares^12^, ^13^ and resulting in the loss of millions of native animals.^14^ The koala (*Phascolarctos cinereus*) was one of the most impacted species,^14^, ^15^, ^16^, ^17^, ^18^ with koala deaths estimated in the range of 40,000 to 50,000 in South Australia (SA) alone.^14^, ^15^, ^16^, ^17^, ^18^, ^19^ In New South Wales (NSW) and Victoria (VIC), 5.7 million and 1.6 million hectares of land were burnt respectively,^20^ resulting in the loss of multiple koala colonies.^21^ In addition to the direct loss of koalas directly impacted by the wildfires, hundreds of koalas which survived (termed here as fire‐affected) were taken into care at wildlife and veterinary hospitals and treated for burn wounds, injuries, disease, starvation and/or dehydration,^15^, ^17^, ^18^ with the aim of rehabilitation and release.

Fire‐affected koalas typically outwardly present with cutaneous burn wounds to foot pads, limbs and the face.^15^, ^18^, ^22^ Burn wound infections and sepsis are reported as major causes of morbidity and mortality in wildlife undergoing treatment for cutaneous burn injuries.^23^ Burnt koalas may also acquire smoke inhalation and thermal injuries to the upper and lower respiratory tract, which can cause pulmonary oedema, pneumonia and respiratory failure.^24^, ^25^

Currently, there are no published studies examining bacterial colonisation and infection of cutaneous burn wounds in wildlife specifically; however, rodent and porcine burn models are frequently used in research studies.^26^, ^27^ In human and animal model studies, immediately after a cutaneous burn injury, the wound is usually sterile; however, gram‐positive bacteria from normal skin flora, most commonly *Staphylococcus aureus*, rapidly establish in the wound. Subsequently, gram‐negative bacteria from the environment and/or gut microbiome, most commonly *Pseudomonas aeruginosa*, then colonise the wound.^28^, ^29^ The colonisation of burn wounds by bacteria alone does not constitute infection; however, without treatment, colonisation may rapidly progress to infection.^30^ In human patients, burn wound infection has been defined as the presence of >10^5^ bacteria per gram of tissue, in combination with clinical signs including cellulitis, erythema, increased warmth, pain, exudative drainage, wound colour changes, odour, invasion into unburned tissue and frequently systemic sepsis.^28^, ^30^, ^31^

Along with coagulase‐positive *Staphylococcus spp*., other common gram‐positive pathogens found in burn wound infections are coagulase‐negative staphylococci, *Streptococcus pyogenes* and *Enterococcus* spp.^32^ In addition to *Pseudomonas aeruginosa*, other frequent gram‐negative pathogens include *Escherichia coli, Klebsiella pneumoniae, Enterobacter species, Proteus* spp. and *Acinetobacter baumannii*.^32^, ^33^ Anaerobic bacteria are infrequently associated with burn wound infections but can include *Bacteroides* spp., *Clostridium* spp. and *Fusobacterium* spp.^32^

In burns treatment protocols for human and wildlife patients, the key recommendation is to prevent cutaneous burn wounds from becoming infected.^28^, ^34^ Preventive methods include early surgical debridement, topical antibiotics and sterile dressings and/or bandages.^28^, ^34^ Prophylactic systemic antibiotic administration for noninfected cutaneous burn wounds is not recommended in humans or wildlife,^28^, ^34^ as published evidence indicates that systemic antibiotics do not reduce infection rates.^35^ However, koalas with burn wounds may also develop clinical bacterial infections that do require antibiotic treatment, including cellulitis, osteomyelitis, pneumonia, sepsis^34^ or chlamydial disease, which is a significant health threat for koalas.^36^ In koalas, chlamydial disease is caused by *Chlamydia pecorum* ^37^ and the first‐choice antibiotic treatments are injectable chloramphenicol or long‐acting doxycycline.^38^, ^39^, ^40^, ^41^, ^42^

Oral and systemic antibiotic administration to koalas is inherently high risk due to the sensitivity of the koala gut microbiome.^43^ Use of antibiotics in koalas has been shown to induce composition shifts in the koala gut microbiome, including a reduced abundance of specialised gut bacteria essential for digesting eucalypt leaves.^44^ The disruption to the koala microbiome may lead to intestinal dysfunction, emaciation and death, a syndrome referred to as fatal gastrointestinal dysbiosis.^43^ The risks associated with antibiotic therapy for koalas require antibiotics to be administered judiciously and conservatively, and when they are used, the clinical indication should be clear and bacterial infection confirmed.^43^

During the catastrophic wildfires of 2019–2020, wildlife veterinarians and rehabilitators who responded indicated that large quantities of antibiotics may have been administered to fire‐affected wildlife due to the unprecedented wildlife emergency and wide range of responders operating under stressful conditions. Hence, we had some concerns regarding inappropriate antibiotic usage that could have negatively impacted Australian wildlife, further threatening the health and outcomes for the fire‐affected animals.

Here, we undertook a retrospective analysis of data from fire‐affected koalas treated at two wildlife/veterinary hospitals during and after the 2019–2020 wildfires in Australia. Triage and treatment records were analysed to determine how frequently antibiotics were administered to fire‐affected koalas, why they were administered, and if established treatment protocols and antimicrobial prescribing guidelines were followed.

## Materials and methods

### Koala triage and treatment records

Triage and treatment records were obtained for 355 fire‐affected koalas admitted into care at two koala treatment facilities in Australia (hereon referred to as ‘Facility A' and ‘Facility B'). Of the 355 koalas, 315 were admitted between 9 January 2020 and 15 March 2020 to Facility A, and 40 were admitted between 2 November 2019 and 9 December 2019 to Facility B.

### Triage and treatment record data

Each koala was assigned a unique identification number and/or name at admission and underwent triage assessment. Clinical examination records included the koala's estimated age, body condition, body weight, sex, presence of joey, any abnormalities detected (including cutaneous burn wounds, nonburn wounds, infections, dehydration and neurological signs) and a treatment plan.

Ongoing treatment records for each koala included repeat clinical examination/assessment notes, details of treatments performed (including anaesthesia, treatment of burn wounds and other injuries/infections), administered medications, clinical tests performed and test results (including chlamydial disease status) and an ongoing treatment plan. The final outcome of each koala, that is, euthanasia, death, released, escaped, transferred or retained in captivity, was also recorded in treatment records. Based on triage and treatment records, the health status of koalas was assigned to one of six categories: Cutaneous burn wounds present; Cutaneous burn wounds and chlamydial disease present; Non‐burns related injuries and/or illness (excluding chlamydial disease); Chlamydial disease only; No detectable injuries and/or illness; Unknown health status (insufficient data recorded).

### Administration of systemic antibiotics

Koala triage and treatment records were examined for the administration of systemic antibiotics during treatment. Systemic administration was reported as subcutaneous injection (SC or SQ), intramuscular injection (IM), intravenous injection (IV) or oral administration (PO). Koalas were placed into one of two categories: koalas that received systemic antibiotics and koalas that did not receive systemic antibiotics. For those koalas that did receive systemic antibiotics, the number of different antibiotic types administered to individual koalas was tabulated, and the reason for the use of multiple antibiotic types was determined from triage and treatment notes.

### Classification of cutaneous burns

For koalas receiving systemic antibiotics, cutaneous burn wounds were classified according to the descriptive terms used in triage and treatment records, and categorised as either No burn wounds identified (Category 1); Mild to moderate burn wounds not requiring bandaging (Category 2) (keywords; mild, moderate, minor, superficial, partial thickness, singed, scab, healing/healed); or Moderate to severe burn wounds requiring bandaging (Category 3) (keywords; severe, full thickness, extensive, deep, radiant, ulcerated, eschar, skin loss, necrotic).

### Clinical indications for antibiotic administration

The clinical indication for initiating antibiotic treatments was determined from koala triage and treatment records. Each antibiotic treatment was assigned to one of six categories: Infections associated with cutaneous burn wounds; Chlamydial infections; Other infections (i.e., non‐burn wounds and non‐chlamydial infections); Category 3 burns reported as noninfected; Category 2 burn wounds reported as noninfected; No infection identified (i.e. no clinical indication).

As tissue biopsy, histology and culture and susceptibility testing were not routinely performed, infections were deemed likely to be present by clinic signs and the use of specific descriptive terms in triage and treatment records: infection, purulent, pus, discharge, abscess, cellulitis, swelling/swollen, festy, deep wound/laceration, non‐healing wound, diarrhoea, lesions and dermatitis. Where these descriptive terms were absent in treatment records, burn wounds were deemed to be reported as noninfected and/or no other infections were deemed to be present.

Chlamydial disease status data were provided for 111 of 315 koalas at Facility A (diagnostic qPCR testing of urogenital swabs) and all 40 koalas at Facility B (clinical signs, ultrasound findings and/or diagnostic testing of urogenital and/or ocular swabs by PCR).

### Antibiotic treatment dosages

Where complete treatment records (from triage to koala outcome) were available, the length of antibiotic treatment periods, antibiotic dose and the number of administered and missed antibiotic doses were calculated. A treatment period including multiple antibiotics belonging to the same class was considered one treatment period, whereas switching treatment to a different antibiotic class was considered a new treatment period. Administered doses were calculated for each koala using recorded body weights, volume of drug administered and the concentration of antibiotic solution. Where the dose varied across the treatment period for an individual koala, the average dose was calculated. All doses and treatment period lengths are reported as mean ± SEM. A literature search was undertaken to determine the recommended dose for each administered antibiotic type.

### Data analysis and statistical analyses

All data analysis and descriptive statistics for doses and antibiotic treatment lengths were performed using Microsoft Excel (Microsoft, Redmond, USA).

## Results

### Systemic antibiotics administered to admitted koalas and recommended doses

A total of eight antibiotics were administered to fire‐affected koalas admitted to the two koala treatment facilities during the 2019–2020 wildfires (Table 1). These belonged to six classes of antibiotics: penicillins, cephalosporins, fluoroquinolones, phenicols, tetracyclines and folate pathway inhibitors (Table 1). The specific antibiotic products used, wildlife/veterinary hospital recommended doses and published recommended doses are summarised in Table 1.

**Table 1:** Antibiotic (and its class), product trade name and manufacturer, recommended doses and references for suggested doses of antibiotics used in the treatment of fire‐affected koalas during the 2019–2020 wildfires at two koala treatment facilities (Facility A and Facility B)

Antibiotic (antibiotic class)	Product trade names (manufacturer)	Antibiotic spectrum of activity	Wildlife/veterinary hospital recommended doses	Published recommended doses for koalas
Long‐acting amoxicillin (penicillin)	Betamox L.A. Injection (Norbrook)	Broad spectrum against gram‐positive and some gram‐negative bacteria. Less activity than narrow spectrum penicillins against anaerobes. No activity against *Pseudomonas aeruginosa, Klebsiella pneumoniae* or methicillin‐resistant *Staphylococcus* spp.	Facility A: 10–15 January 2020: 15 mg/kg SC q48h 4‐6d From 16 January 2020: 15 mg/kg SC SID 3‐5d	Long‐acting amoxicillin – not available Amoxicillin – 12.5 mg/kg SC BID (PK)^45^
Amoxicillin‐clavulanic acid (penicillin + beta‐lactamase inhibitor)	Noroclav injection (Norbrook) Clavulox injection (Zoetis)	Broad spectrum against gram‐positive and some gram‐negative bacteria. Less activity than narrow spectrum penicillins against anaerobes. No activity against *Pseudomonas* aeruginosa or methicillin‐resistant *Staphylococcus* spp.	Facility A: 8.75 mg/kg SC SID 3‐5d Facility B: Not provided on triage/treatment forms	12.5 mg/kg SC/IM BID (EM, PK)^45^, ^46^
Procaine benzylpenicillin and benzathine benzylpenicillin (penicillin)	Duplocillin (MSD animal health)	Narrow spectrum against gram‐positive and anaerobic bacteria. No activity against *Pseudomonas aeruginosa, Klebsiella pneumoniae* or methicillin‐resistant *Staphylococcus* spp.	Facility A: Not provided on triage/treatment forms	26.5 mg/kg SC/IM q72h (long‐acting) (EM)^47^
Ceftazidime (cephalosporin – third generation)	Ceftazidime 1 g (Sandoz or Viatris)	Reserved to treat *Pseudomonas aeruginosa*.	Facility A: Not provided on triage/treatment forms	20 mg/kg IV q8h (EM)^46^
Enrofloxacin (fluoroquinolone)	Baytril (Bayer), Ilium Enrotril (Troy)	Activity against many gram‐negative and gram‐positive bacteria. Ineffective against anaerobic bacteria. Includes some activity against *Pseudomonas aeruginosa* and *Staphylococcus* spp.	Facility A: Not provided on triage/treatment forms Facility B: 10mg/kg SC SID 14d	10 mg/kg SC SID (chlamydial disease) (PK)^48^ 8–10 mg/kg SC/IV SID/BID (infection) (EM)^46^
Chloramphenicol (Phenicol)	Chloramphenicol 150 (Ceva animal health)	Broad spectrum of activity against gram‐positive and gram‐negative, aerobic and anaerobic bacteria. Activity against *Chlamydia* spp. poor activity against *Pseudomonas aeruginosa*.	Facility B: 60 mg/kg SC SID 21‐28d	60 mg/kg SC SID 28‐45d (PK, EM)^42^, ^46^ 60 mg/kg SC SID 14‐28d (EM)^41^
Doxycycline (tetracycline)	Doxycycline LA (Vetafarm)	Broad spectrum of activity against gram‐positive and gram‐negative, aerobic and anaerobic bacteria. Activity against *Chlamydia* spp. not indicated to inhibit *Pseudomonas aeruginosa*.	Facility A: Not provided on triage/treatment forms	5 mg/kg SC q7d 28‐42d (long‐acting doxycycline for chlamydial disease) (EM)^38^, ^39^
Trimethoprim and sulfamethoxazole (folate pathway inhibitor)	Septrin (Wellcome Australia)	Broad spectrum of activity against gram‐negative bacteria and gram‐positives (including some methicillin‐resistant *Staphylococcus aureus*). Not active against anerobic bacteria *in vivo*, nor *Pseudomonas aeruginosa*.	Facility A: Not provided on triage/treatment forms	15 mg/kg PO BID (EM)^46^

No listed antibiotics are registered for use in koalas. All listed antibiotics (except ceftazidime) are registered for use in companion animals.

Abbreviations: BID, twice per day; d, days; EM, empirical determined dose; h, hours; IM, intramuscular injection; IV, intravenous injection; PK, pharmacokinetic study determined dose; PO, per os (oral administration); q48h, every 48 hours; q72h, every 72 hours; q7d, every 7 days; q8h, every 8 hours; SC, subcutaneous injection; SID, once per day.

### Analysis of triage and/or treatment records of admitted koalas

Analysis of triage/treatment records of koalas admitted to Facility A (*n* = 315) and Facility B (*n* = 40) during the 2019–2020 wildfires revealed that 69.6% (247/355) of all admitted koalas presented with burn wounds (Table 2). Overall, 10.6% of tested koalas (16/151) had chlamydial disease, with all detections at Facility B (Table 2). Other injury or illness was reported in 11.3% (40/355) of koalas, and 17.2% (61/355) of koalas presented with no detectable abnormalities (rescued from burnt habitat) (Table 2). Koalas with cutaneous burn wounds were treated using aseptic cleansing, topical antiseptics/antibiotics (including chlorhexidine, povidone‐iodine and/or silver sulfadiazine), surgical debridement and bandaging of category 3 burn wounds to feet and/or limbs. No treatment records reported koalas presenting with pneumonia or respiratory failure.

**Table 2:** Summary of health status data from triage/treatment records of 355 koalas admitted to two koala treatment facilities during the 2019–2020 wildfires

Koala health status category	Facility A (*n* = 315)	Facility B (*n* = 40)	Total (*n* = 355)
Cutaneous burn wounds present	223 (70.8%)	24 (60.0%)^b^	247 (69.6%)
Non‐burns injuries/illness present (excluding chlamydial disease)	37 (11.7%)	3 (7.5%)	40 (11.3%)
Chlamydial disease	0 (0.0%)^a^	16 (40.0%)^b^	16 (10.6%)^a^
No detectable injuries/illness	50 (15.9%)	11 (27.5%)	61 (17.2%)
Unknown health status	5 (1.6%)	0 (0.0%)	5 (1.4%)

^a^ Urogenital swabs were collected for chlamydial disease diagnostic qPCR testing from 111 of 315 koalas admitted to Facility A, that is, 0/111 (0.0%) koalas were chlamydia positive at Facility A, and 16/151 (10.6%) were chlamydia positive overall.

^b^ At Facility B, 14 koalas presented with both cutaneous burn wounds and chlamydial disease.

### Systemic antibiotics administered to koalas treated at two koala treatment facilities

At least one dose of systemic antibiotics was administered to 40.3% (127/315) of koalas at Facility A and 35.0% (14/40) of koalas at Facility B 39.7% (141/355). Five antibiotics were exclusively administered to koalas at Facility A: long‐acting amoxicillin, ceftazidime, doxycycline, trimethoprim‐sulfamethoxazole and procaine benzylpenicillin‐benzathine benzylpenicillin (Table 3). Chloramphenicol was exclusively administered to koalas at Facility B (Table 3). Enrofloxacin and amoxicillin‐clavulanic acid were administered to koalas treated at both Facility A and Facility B (Table 3). The most frequently administered antibiotics were penicillins (Table 3), with 35.8% (127/355) of all admitted koalas receiving at least one type. Enrofloxacin was the second most frequently administered antibiotic to koalas (7.6%, 27/355) (Table 3).

**Table 3:** Types and frequency of antibiotics administered to 355 koalas admitted to two koala treatment facilities during the 2019–2020 wildfires

Administered antibiotic	Route of administration	Facility A (*n* = 315)	Facility B (*n* = 40)	Total (*n* = 355)
Amoxicillin (long‐acting)	SC	94 (29.8%)	0 (0.0%)	94 (26.5%)
Amoxicillin + clavulanic acid	SC/IM^a^	29 (9.2%)	4 (10.0%)	33 (9.3%)
Procaine benzylpenicillin + benzathine benzylpenicillin	SC	6 (1.9%)	0 (0.0%)	6 (1.7%)
Ceftazidine	IV	1 (0.3%)	0 (0.0%)	1 (0.3%)
Enrofloxacin	SC	18 (5.7%)	9 (22.5%)	27 (7.6%)
Chloramphenicol	SC	0 (0.0%)	6 (15.0%)	6 (1.7%)
Doxycycline	SC	1 (0.3%)	0 (0.0%)	1 (0.3%)
Trimethoprim + sulfamethoxazole	PO	1 (0.3%)	0 (0.0%)	1 (0.3%)

^a^ Three doses of amoxicillin +/− clavulanic acid were given IM (all other were SC).

Abbreviations: IM, intramuscular injection; IV, intravenous injection; PO, per os (oral administration); SC, subcutaneous injection.

### Number of antibiotic types administered to individual koalas

Of the 141 koalas receiving antibiotics, the majority were administered one type of antibiotic (74.5%, 105/141), with 23.4% (33/141) receiving two different antibiotics and 2.1% (3/141) receiving three different antibiotics (Table S1). The vast majority of koalas administered multiple antibiotics received sequential treatments (35/36) and only one koala (at Facility A) received multiple concurrent antibiotics (Table S1). A similar proportion of koalas at both treatment facilities received multiple antibiotics (Facility A 24.4% and Facility B 35.7%); however, the combinations of antibiotics administered differed (Table S1). The two most frequent combinations used in koalas at Facility A were amoxicillin +/− clavulanic acid and enrofloxacin (11.8%, 15/127) or multiple types of penicillins (14.2%, 14/127) (Table S1). Whereas the two most frequent combinations administered to koalas at Facility B were chloramphenicol and enrofloxacin (28.6%, 4/14) or amoxicillin‐clavulanic acid and enrofloxacin (7.1%, 1/14) (Table S1).

At Facility A, 13 koalas were switched to a different antibiotic after the initial triage assessment and treatment. These changes in antibiotics were related to the introduction of long‐acting amoxicillin to the facility on 12 January 2020, 2 days after treatments commenced. Koalas triaged before 12 January predominantly received amoxicillin‐clavulanic acid (*n* = 27) and six received procaine benzylpenicillin‐benzathine benzylpenicillin. Antibiotic treatments were also changed in 21 koalas at Facility A due to infection developing or being considered nonresponsive to the current antibiotic. At Facility B, four koalas initially received enrofloxacin for chlamydial disease treatment and were subsequently changed to chloramphenicol. Treatment records for these koalas reported that injectable chloramphenicol was in short supply or unavailable during November 2019 and December 2019, and enrofloxacin was used as an alternative (second‐choice) treatment when chloramphenicol (first choice) was unavailable.

### Clinical indications for systemic antibiotic treatment

**Figure 1:**
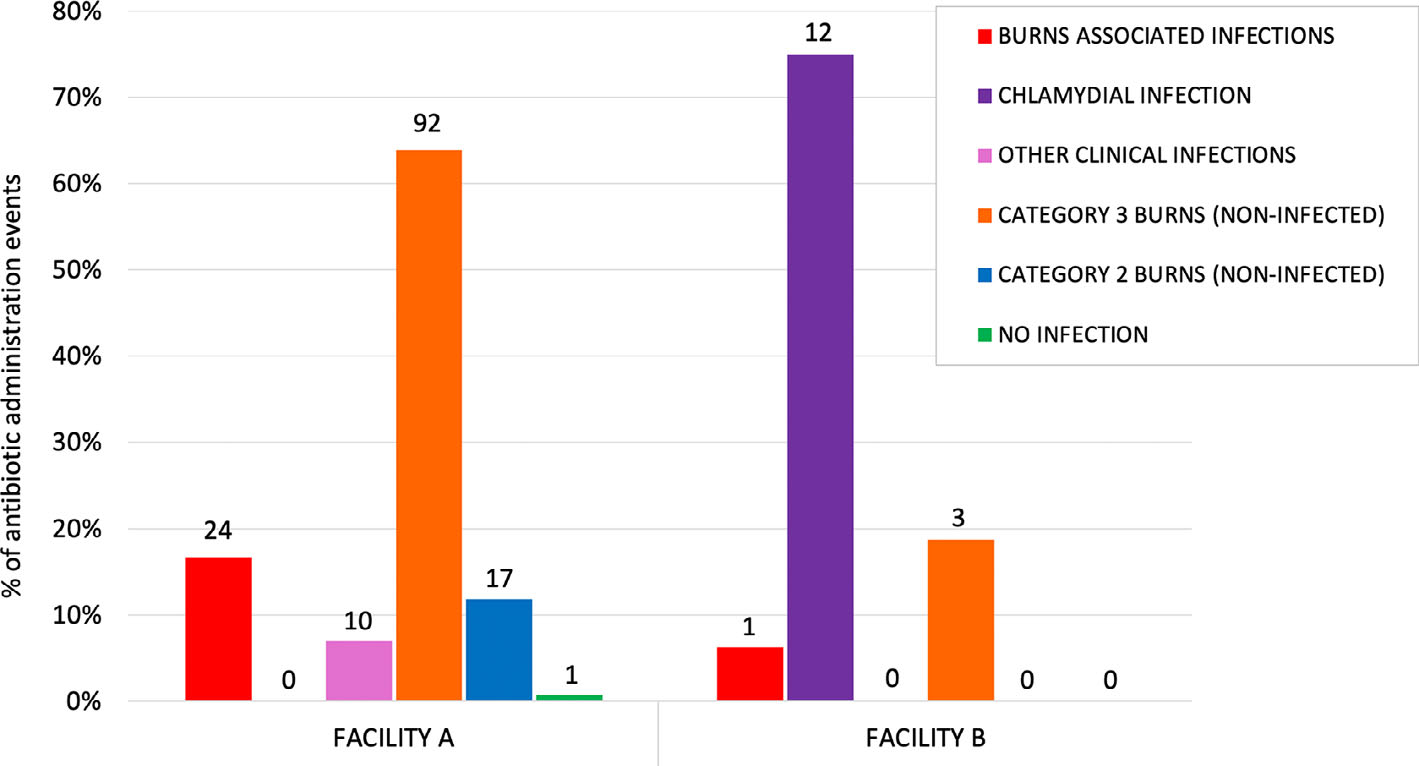
Clinical indications for 160 systemic antibiotic treatment events administered to 141 koalas during the 2019–2020 wildfires at two treatment facilities. Numbers above the bars indicate the number of treatment events.

Of the 141 koalas receiving systemic antibiotics, 19 received antibiotics on two separate occasions, resulting in a total of 160 antibiotic treatment events (Facility A, *n* = 144 treatment events; Facility B, *n* = 16 treatment events). The clinical indication for commencing systemic antibiotic administration for all 160 treatment events was determined from treatment records and assigned to one of six categories (Figure 1).

At Facility A, the majority of systemic antibiotic treatment events (63.9%, 92/144) were prophylactic administration for noninfected Category 3 burn wounds (moderate to severe requiring bandaging) (Figure 1). Overall, 23.6% (34/144) antibiotic treatment events at Facility A were administered for clinical infections, including infections associated with cutaneous burn wounds and the respiratory tract (Figure 1).

At Facility B, the predominant clinical indication for systemic antibiotic administration was for the treatment of chlamydial disease (75.0%, 12/16 treatment events), followed by prophylactic treatment of noninfected Category 3 burn wounds (moderate to severe requiring bandaging) (18.8%, 3/16 treatment events) and 1 koala (6.3%) received antibiotics for infected burns (Figure 1).

### Types of antibiotics administered for different clinical indications

**Figure 2:**
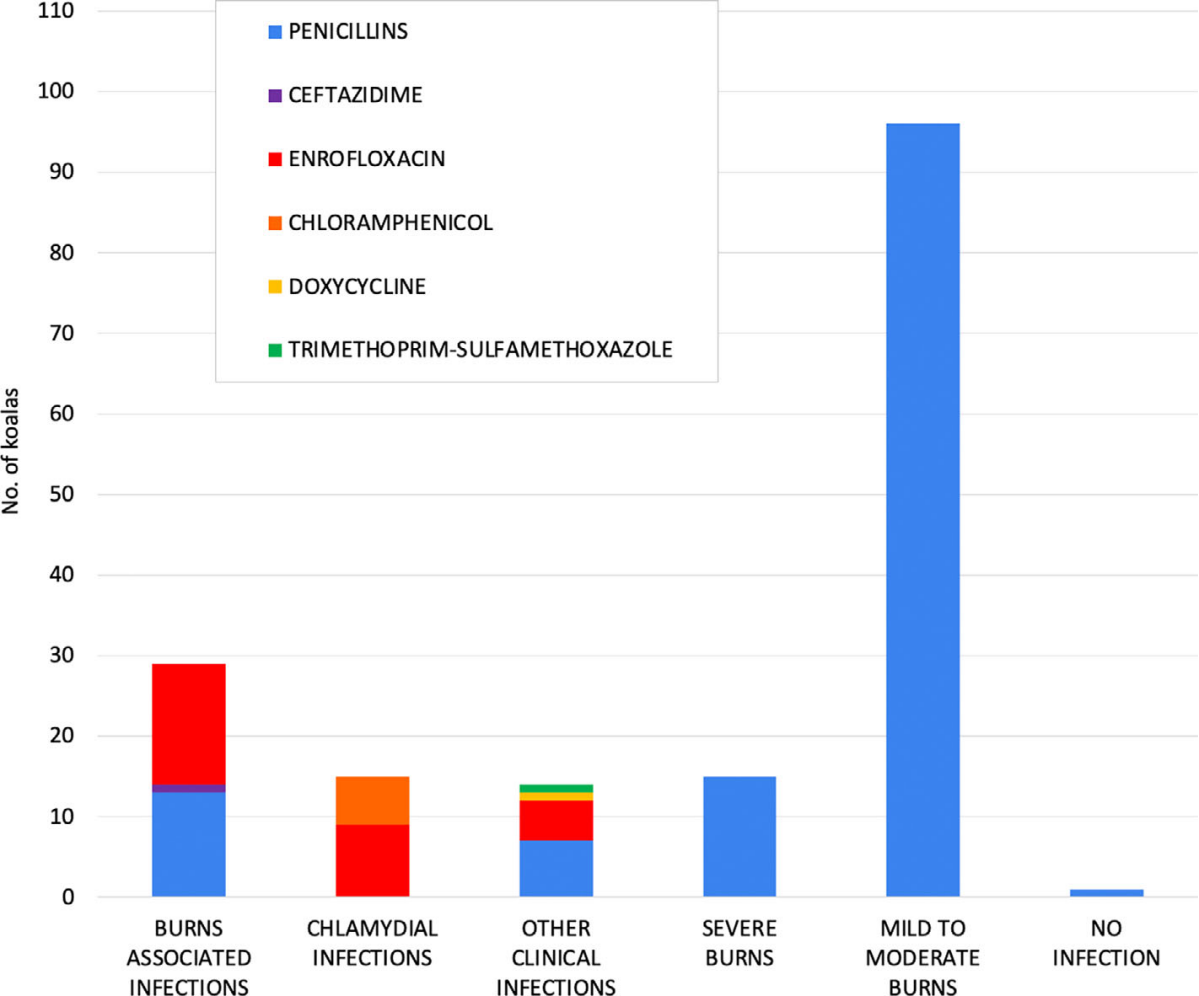
Types of antibiotics administered to koalas for different clinical indications during the 2019–2020 wildfires.

Penicillins (long‐acting amoxicillin, amoxicillin‐clavulanic acid and procaine benzylpenicillin‐benzathine benzylpenicillin) were predominantly administered as prophylactic treatment to koalas with noninfected Category 3 burn wounds (*n* = 95) (Figure 2). Penicillins (*n* = 19) and/or enrofloxacin (*n* = 19) were both administered to koalas with clinical infections, excluding chlamydial disease (Figure 2). Koalas treated for chlamydial disease were administered chloramphenicol (*n* = 6) and/or enrofloxacin (*n* = 9), depending on chloramphenicol availability (Figure 2).

### Doses of administered antibiotics

**Figure 3:**
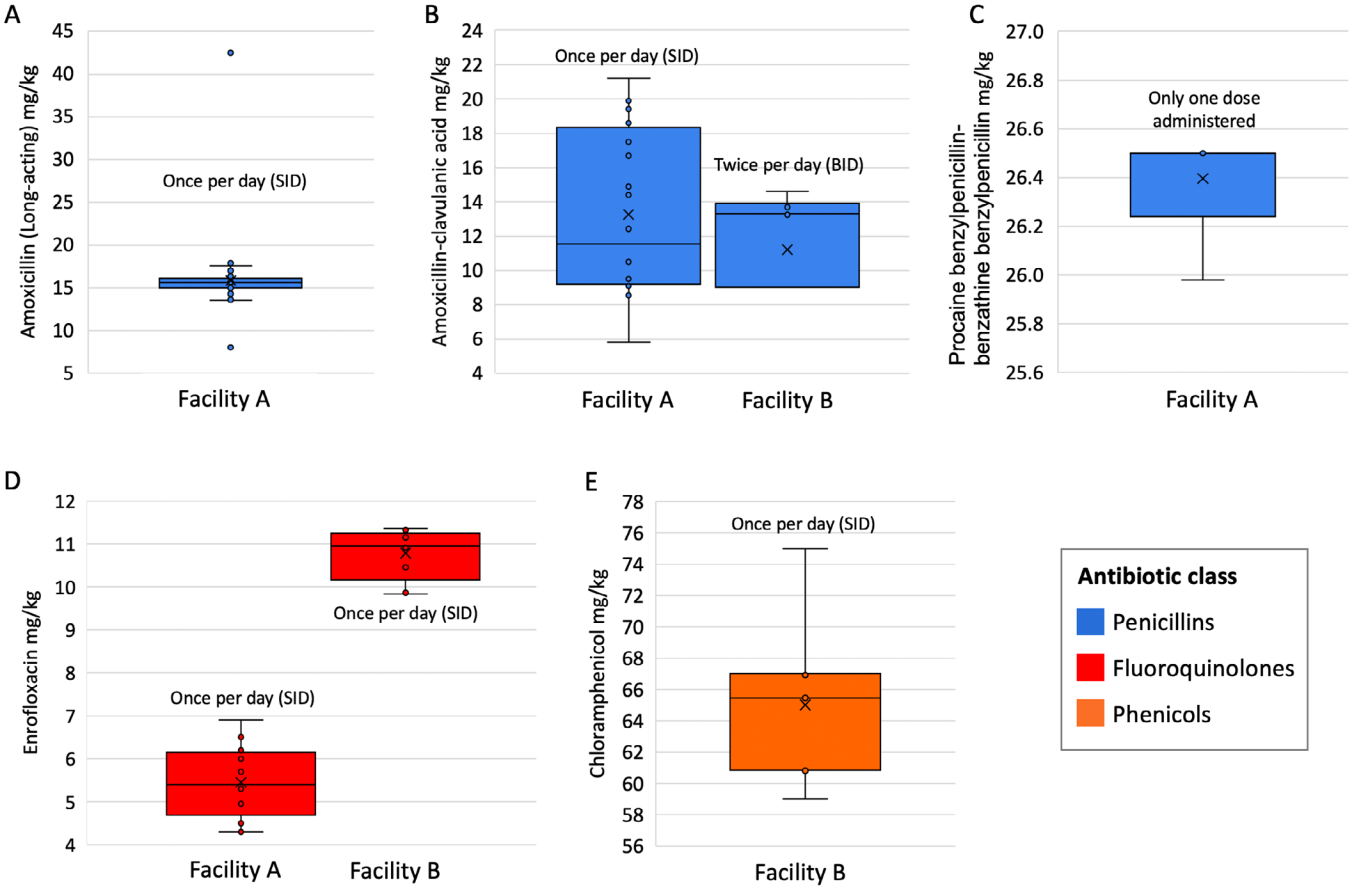
Box and whisker plots displaying the doses for five antibiotics administered to koalas at the two treatment facilities during the 2019–2020 wildfires. Boxes indicate the interquartile ranges. X indicates the mean dose. (A) Long‐acting Amoxicillin. (B) Amoxicillin‐clavulanic acid. (C) Procaine benzylpenicillin‐benzathine benzylpenicillin. (D) Enrofloxacin. (E) Chloramphenicol.

Complete triage/treatment records with antibiotics doses were available for 111 of the 141 koalas that received systemic antibiotics. All doses are reported as mean ± SEM. The mean dose of long‐acting amoxicillin administered to koalas at Facility A was 15.8 ± 0.34 mg/kg SC (range 13.5–17.9 mg/kg SC, excluding two outliers) (Figure 3(A)), which is in line with the recommended dose provided on the Facility A triage form (15 mg/kg SC) (Table 1). It should be noted that between 9 and 15 January, the recommended dose was 15 mg/kg SC every 48 h; however, from 16 January onwards, the recommended frequency was increased to every 24 h. Currently, there are no published recommended doses for long‐acting amoxicillin administration in koalas; however, the recommended dose for amoxicillin (non‐long‐acting) is 12.5 mg/kg SC twice per day (Table 1).

The mean dose of amoxicillin‐clavulanic acid administered to Facility A koalas was 13.3 ± 1.07 mg/kg SC once per day (range 5.8–21.2 mg/kg) (Figure 3(B)), which was higher than the recommended dose provided on the Facility A triage form (8.75 mg/kg SC once per day) (Table 1). Koalas at Facility B were administered a similar mean dose of 13.4 ± 0.58 mg/kg; however, they were dosed twice per day (Figure 3(B)). The administered mean dose of procaine benzylpenicillin‐benzathine benzylpenicillin was 26.4 ± 0.09 mg/kg (Figure 3(C)), which was comparable with the published recommended dose of 26.5 mg/kg (Table 1).

The Facility B koalas mean dose for enrofloxacin (10.8 +/− 0.20 mg/kg SC once per day) was comparable with the published recommended dose (8–10 mg/kg SC/IV once per day), whereas Facility A koalas received a much lower mean dose (5.5 +/− 0.20 mg/kg SC once per day), which is below the recommended dose (Figure 3(D) and Table 1). The mean dose of chloramphenicol administered to koalas at Facility B was 65.0 ± 2.06 mg/kg SC once per day (Figure 3(E)) and comparable with the recommended dose of 60 mg/kg SC once per day (Table 1). Ceftazidime was administered to one koala at 21.0 mg/kg IV three times per day, which is comparable with the published dose of 20.0 mg/kg IV three times per day (Table 1). Insufficient data were available to calculate the administered doxycycline and trimethoprim‐sulfamethoxazole doses.

### Length of antibiotic treatment

For all koalas with complete triage and/or treatment records (*n* = 111), the length of all antibiotic treatment periods (*n* = 130) was determined. Treatment period lengths are reported as mean ± SEM. The mean length of electively discontinued antibiotic treatment periods was 5.3 ± 0.90 days (*n* = 40, range 1–31 days) in koalas at Facility A and 14.1 ± 1.52 days (*n* = 20, range 5–28 days) in koalas at Facility B. The majority of antibiotic treatments in koalas at Facility A were discontinued due to death, euthanasia or escape (63.6%, 70/110), of which the mean treatment length was 4.3 ± 0.39 days (range 1–13 days).

In koalas at facility A, 20.9% (23/110) of all antibiotic treatments commenced were electively discontinued after 1–4 days, 37.3% (41/110) were discontinued after 1–4 days due to death or escape and 40.0% (44/110) of all antibiotic treatments were 5 or more days in length (Table 4). For koalas at facility B, 100% (20/20) of antibiotic treatments were 5 or more days in length (Table 4). Antibiotic treatments for chlamydial disease had longer treatment periods (range 6–28 days) compared with koalas receiving amoxicillin‐clavulanic acid administration for burn wounds (range 5–7 days) at facility B.

**Table 4:** Lengths of antibiotic treatment periods (*n* = 130) for koalas during the 2019–2020 wildfires at two facilities

Treatment length	Facility A (*n* = 110)	Facility B (*n* = 20)	Total (*n* = 130)
Treatment period 1–2 days (electively discontinued)	14 (12.7%)	0 (0.0%)	14 (10.8%)
Treatment period 3–4 days (electively discontinued)	9 (8.2%)	0 (0.0%)	9 (6.9%)
Treatment period 5–10 days	35 (31.8%)	7 (35.0%)^a^	42 (32.3%)
Treatment period ≥11 days	9 (8.2%)	13 (65.0%)^a^	22 (16.9%)
Treatment period 1–4 days (death/euthanasia/escape)	41 (37.3%)	0 (0.0%)	41 (31.5%)
Treatment period <5 days (changed antibiotic class)	2 (1.8%)	0 (0.0%)	2 (1.5%)

^a^ Chloramphenicol treatment periods for three koalas at facility B were stopped early (after 9–12 days, instead of the planned 21–28 days) due to inadequate supply of injectable chloramphenicol.

A treatment period including multiple penicillin antibiotic types is considered one treatment period.

Switching treatment to a different antibiotic class is considered a new treatment period.

### Missed or unrecorded antibiotic doses during treatment period

The number of scheduled antibiotic doses that were missed or not recorded as administered during each antibiotic treatment period was determined for all koalas with complete triage and/or treatment records (*n* = 111). In koalas at Facility A, 26.0% (134/515) of scheduled antibiotic doses were missed or not recorded as administered (Figure S1a,b). The high proportion of missed and/or unrecorded antibiotic doses in Facility A koalas largely correlates with koalas being re‐examined and treated by veterinarians every 2–3 days, usually for bandage changes. In koalas admitted to Facility B, 1.0% (3/306) of scheduled antibiotic doses were missed or not recorded as administered (Figure S1c).

### Outcome of koalas receiving antibiotics

Outcome was recorded for 127 koalas that received systemic antibiotics at Facility A and 14 at Facility B. At Facility A, 63.8% (81/127) were euthanised or died, 12.6% (16/127) were released and 23.6% (30/127) were recorded as unknown outcome (Table S2). At Facility B, 50% (7/14) koalas were either euthanased or died, 35.7% (5/14) were released and 1.4% (2/14) were considered not suitable for release and retained in captivity (Table S2).

## Discussion

This research demonstrates the importance of preparedness for wildlife emergency responses during fires, such as the catastrophic 2019–2020 wildfires in Australia. The study identified significant knowledge gaps regarding appropriate antibiotic use and stewardship in veterinarians that had limited access to established treatment guidelines and/or were inexperienced in treating fire‐affected koalas and other wildlife.

The unprecedented wildfires and high numbers of injured wildlife required the establishment of wildlife emergency response centres, such as Facility A, to triage the overwhelming number of burnt koalas requiring veterinary care. An ongoing fire threat to the facility and the absence of preplanning and infrastructure (including internet and phone connections) meant access to koala burns treatment protocols and published antibiotic doses by treating veterinarians was extremely difficult in the early stages of the response. The absence of digital medical record keeping, continual turnover of volunteer veterinarians and minimal opportunity to fully induct and supervise new veterinarians on arrival also contributed to reduced record keeping, interrupted communication between personnel and discontinuity of care. Consequently, systemic antibiotic prescribing was frequently not in line with burns treatment protocols and published antibiotic doses for koalas.^49^, ^50^, ^51^ In particular, there was an overuse of prophylactic systemic antibiotics in koalas with moderate to severe burn wounds reported as noninfected, which is contradictory to recommended burns protocols for humans^28^, ^35^ and the current treatment protocols for koalas.^25^, ^34^, ^43^, ^49^

For fire‐affected koalas that were treated using established protocols for cutaneous burns and chlamydial disease,^49^ systemic antibiotic administration was largely in line with available treatment protocols and published antibiotic doses. However, best practice antibiotic prescribing was not always feasible due to shortages or unavailability of first‐choice antibiotics. The high number of fire‐affected koalas with chlamydial disease, combined with a shortage of injectable chloramphenicol (first‐choice antibiotic), resulted in enrofloxacin being used as a second‐choice treatment for many koalas at Facility B.^38^, ^48^

For the majority of koalas that received systemic antibiotic treatment for burn wounds (prophylactic and infected), the choice of antibiotic was probably inappropriate as amoxicillin +/− clavulanic acid is ineffective against *Pseudomonas aeruginosa*, that is, the most common gram‐negative pathogen associated with burn wound infections.^32^ Inappropriate antibiotic choice can result in the failure to kill key pathogens commonly associated with burn wound infections and may also inadvertently select for resistant bacterial pathogens.^25^, ^28^ The prophylactic antibiotic treatments administered to burnt koalas were also frequently sub‐optimal relative to dose and length of administration. Sub‐optimal antibiotic administration may lead to inadequate blood concentrations and a failure to kill susceptible bacteria that have the potential to cause infections.^45^, ^48^ Inappropriate antibiotic choice, significant underdosing, inconsistent dosing and short treatment periods may also contribute to the emergence and spread of AMR.^1^

Koalas have numerous anatomical and physiological adaptations for detoxifying their exclusively *Eucalyptus* spp. diet, which may also affect how they metabolise medications, including some antibiotics.^52^, ^53^ Although a number of pharmacokinetic studies have been published on antibiotics in koalas,^45^, ^52^ a pharmacokinetic study on long‐acting amoxicillin, the most frequently administered antibiotic to koalas at Facility A, is lacking. For long‐acting amoxicillin and amoxicillin‐clavulanic acid, Facility A triage forms initially recommended the same doses listed for cats and dogs, that is, long‐acting amoxicillin 15 mg/kg every 48 h, SC/IM and amoxicillin‐clavulanic acid 8.75 mg/kg every 24 h, SC injection.^54^ However, the recommended dosing frequency for long‐acting amoxicillin was subsequently increased to every 24 h, after extrapolation from a pharmacokinetic profile of a non‐long‐acting formulation of amoxicillin in tammar wallabies (*Macropus eugenii*).^55^ Another study investigating the pharmacokinetic profile of amoxicillin (non‐long‐acting formulation, via SC injection) to koalas found poor absorption and faster metabolism compared with domestic animals.^45^ The same study recommended a higher dose of amoxicillin for koalas (12.5 mg/kg, twice per day),^45^ compared with domestic animals (7 mg/kg once per day, SC or IM).^54^ Koala pharmacokinetic studies for several antibiotics (including amoxicillin and enrofloxacin) have shown that extrapolation of doses for domestic animals will result in significant underdosing in koalas^45^, ^52^ and highlight the complexity of antibiotic therapies in wildlife medicine.

Two of eight antibiotics administered to koalas are rated as high importance and two as Medium Important by ASTAG.^11^ High importance antibiotics are essential treatments against infections, where there is a lack of sufficient therapeutic alternatives, and include fluoroquinolones such as enrofloxacin and third generation cephalosporins such as ceftazidime.^11^ Fluoroquinolones are known to select for fluoroquinolone‐resistant *Salmonella* spp. and *E. coli* in animals, and their use should only be considered when the animal has a serious or life‐threatening infection and no therapeutic alternatives are available.^9^

Of the koalas that received antibiotics, 23 were documented as surviving and either released or retained in captivity. Selective pressure associated with antibiotic administration has the potential to select for populations of resistant bacterial pathogens in koala microbiomes.^56^, ^57^ Additionally, antibiotic administration to animals carrying resistant bacteria has the potential to promote the transfer of resistance genes between bacterial species within the microbiome, thus contributing to the emergence of new antibiotic‐resistant pathogens.^58^ After release, there is opportunity for any resistant pathogens that are being carried by these koalas to be disseminated to other animals via the environment.^59^

The insights gained from this research highlight the importance of forward planning and preparedness for future wildfire events to ensure good antibiotic stewardship when treating fire‐affected wildlife. This includes on‐hand mobile emergency response veterinary hospitals and experienced wildlife veterinarians, readily available evidence‐based standardised protocols for the treatment of fire‐affected wildlife, recommended antibiotic administration guidelines for specific wildlife species and provision for documenting detailed treatment records of individual animals. After the catastrophic wildfires of 2019–2020, Taronga Conservation Society has developed a free online E‐learning module (Assessment, Triage and Treatment of Bushfire‐Affected Wildlife) to provide expert knowledge to veterinarians and veterinary hospital personnel treating fire‐affected wildlife (available at https://taronga.org.au/education/study‐taronga/assessment‐triage‐and‐treatment‐bushfire‐affected‐wildlife).^25^ The 2025 edition of *Current Therapy in Medicine of Australian Mammals* has also incorporated training content to support veterinary first responders.^34^ Additionally, adequate supplies of veterinary equipment and medications, including first‐choice antibiotics for treating koalas with severe burns, infections and chlamydial disease, need to be available and accessible for emergency response wildlife hospitals.

Extending from published treatment guidelines on the treatment of bushfire‐affected wildlife^34^ and antibiotic stewardship in Australian wildlife,^60^ the following recommendations arising from this study have been developed to support veterinarians and other emergency responders during a wildfire response:

1. Adhere to established protocols for the treatment of cutaneous burns in wildlife, especially only using topical antiseptics/antibiotics for noninfected burn wounds.
2. Do not administer prophylactic systemic antibiotics to wildlife with noninfected cutaneous burn wounds.
3. Restrict systemic antibiotic administration to wildlife with burn wound infections and/or other clinical infections, such as cellulitis, osteomyelitis, pneumonia or sepsis.
4. Confirm bacterial infection is present and carefully consider the choice of antibiotic, which should preferably be based on culture and susceptibility testing (Table 5).
5. In the absence of culture and susceptibility testing, identify common bacterial pathogens associated with burn wound infections/osteomyelitis/sepsis/pneumonia and select a broad‐spectrum antibiotic that inhibits key pathogens (Table 5).
6. Use recommended antibiotic doses established for the species being treated and preferably doses that reflect evidence‐based pharmacokinetic research.
7. Avoid (where possible) using antibiotics with no pharmacokinetic evidence for doses in the species being treated.
8. Do not extrapolate antibiotic doses recommended for domestic animals into wildlife species.

**Table 5:** Antibiotic strategy to treat bacterial pathogens associated with burn wounds

	Gram‐positive	Gram‐negative	*Pseudomonas*	Anaerobes
Amoxicillin +/− clavulanic acid	Good activity	Some activity	No activity	Some activity
Penicillin (narrow spectrum)	Good activity	No activity	No activity	Good activity
Ceftazidime^a^	No activity	Some activity	Good activity	No activity
Enrofloxacin^a^	Good activity	Good activity	Some activity	No activity
Chloramphenicol	Some activity	Some activity	No activity	Some activity
Doxycycline	Some activity	Some activity	No activity	Some activity
Gentamicin^b^	Some activity	Good activity	Good activity	No activity
Trimethoprim + sulfamethoxazole	Some activity	Some activity	No activity	No activity

*Note*: Shading reflects colour coding for the 3 categories, i.e., blue = ‘good activity’, purple = ‘some activity’ and grey = “no activity”.

^a^ High importance antimicrobials (ASTAG 2018).

^b^ Only administer gentamicin systemically to animals with good renal function and monitor during treatment. Administer once daily only.

Refer to Table 1 for recommended antibiotic doses and Bodley 2025^46^ for recommended gentamicin dose.

The study has demonstrated issues associated with antimicrobial stewardship in wildlife emergencies. Given that climate change will continue to drive an increased risk of extreme wildfires,^61^, ^62^ it is anticipated that wildlife emergency responses to wildfires will occur more frequently. Emergency preparedness for future wildfire events, including readily available evidence‐based protocols for the treatment of fire‐affected wildlife, recommended antibiotic administration guidelines and adequate antibiotic availability, is now critical. Optimally, access to laboratory diagnostic services, including bacterial culture and susceptibility testing, is also required. Overall, this study reinforces the importance of judicious use of antibiotics and adherence to prescribing guidelines when treating fire‐affected wildlife.

## Funding

The study was funded by the Morris Animal Foundation (Grant No. D21ZO‐507 to Michelle Power).

## Ethics statement

Animal ethics approval was not required for this study. All data was obtained retrospectively from treatment and triage records for koalas admitted to two wildlife treatment facilities.

## Conflicts of interest

None of the authors have a conflic of interest to disclose.

## Supporting information


**Data S1.** All data for individual koalas are provided in a supplementary file (Supplementary Data). Supplementary tables are provided in a supplementary file (Supplementary Tables).

Figure S1.

**Table S1.** **Table S2**.

## Data Availability

The data that supports the findings of this study are available in the supplementary material of this article.
