# Supplementary material for: Systemic antibiotic use in fire‐affected koalas ( Phascolarctos cinereus ) admitted to two wildlife treatment facilities during the 2019–2020 wildfires
Source: Aust Vet J. 2025 Aug 5;103(8):475–86. doi: 10.1111/avj.70006 (PMC12331400; doi:10.1111/avj.70006)
Supplement: Supplementary file 2 — Figure S1. [file AVJ-103-475-s002.pdf]

Supplementary Figure

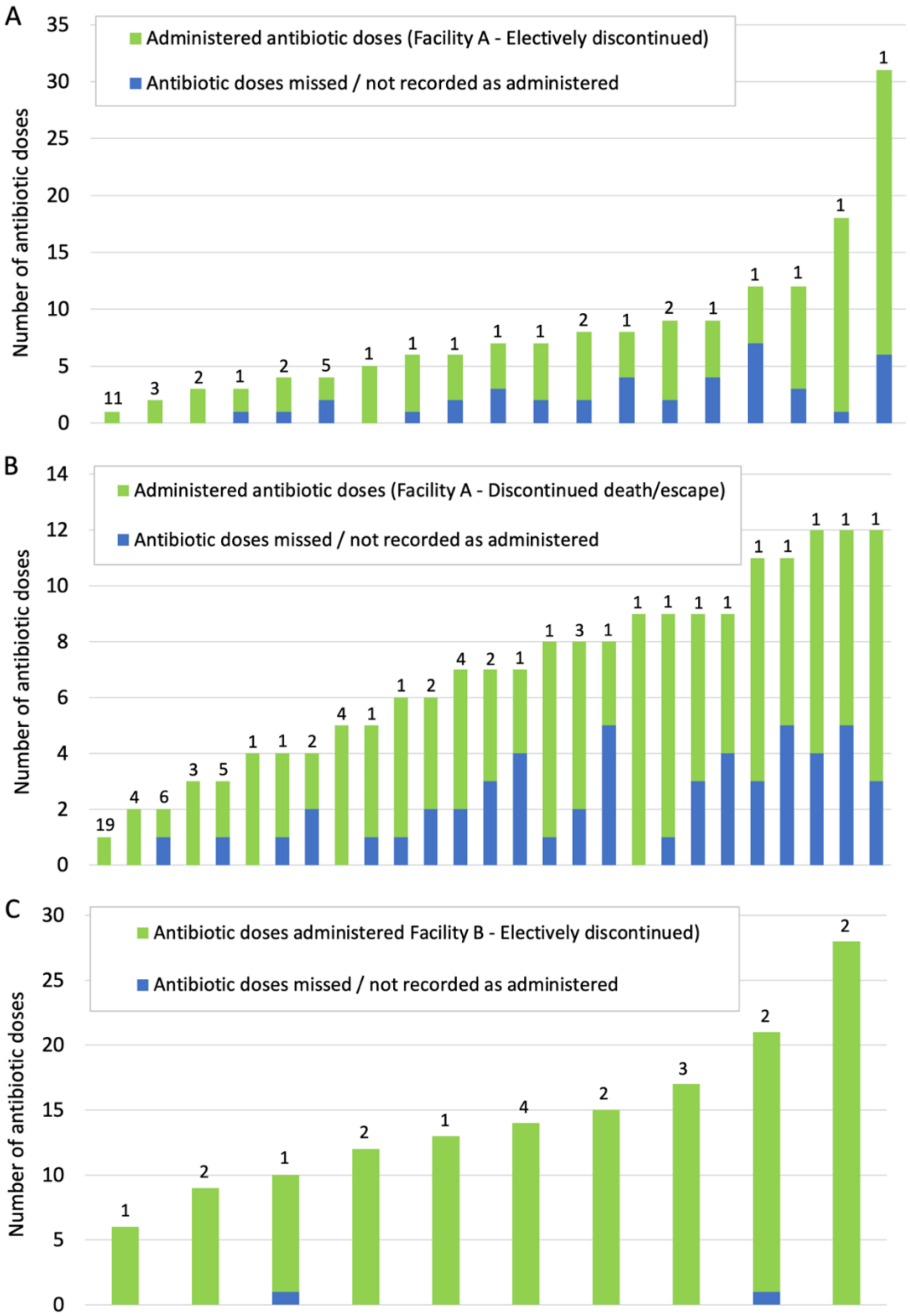

Supplementary Figure 1. Number of administered and missed, or not recorded as administered, antibiotic doses during treatment periods for koalas treated during the 2019-2020 wildfires at the two facilities. A treatment period including multiple penicillin beta-lactam antibiotics is considered one treatment period. Switching treatment to a different antibiotic class is considered a new treatment period. **A**, Electively discontinued treatment periods at Facility A. **B**, Treatment periods discontinued due to death or escape at Facility A. **C**, Electively discontinued treatment periods at Facility B. Numbers above bars indicate the number of koalas per treatment schedule.
