# Supplementary material for: Systemic antibiotic use in fire‐affected koalas ( Phascolarctos cinereus ) admitted to two wildlife treatment facilities during the 2019–2020 wildfires
Source: Aust Vet J. 2025 Aug 5;103(8):475–86. doi: 10.1111/avj.70006 (PMC12331400; doi:10.1111/avj.70006)
Supplement: Supplementary file 3 — Table S1. Table S2. [file AVJ-103-475-s001.pdf]

Supplementary Tables

Supplementary Table 1. Summary table showing antibiotic combinations administered to 355 fire-affected koalas during the 2019/2020 wildfires.

Antibiotic combination	Facility A	Facility B	All koalas
Amoxicillin (long-acting)	51.2% (65/127)	0.0% (0/14)	46.1% (65/141)
Duplocillin	2.4% (3/127)	0.0% (0/14)	2.1% (3/141)
Amoxicillin/clavulanic acid	18.9% (24/127)	21.4% (3/14)	19.1% (27/141)
Enrofloxacin	2.4% (3/127)	28.6% (4/14)	5.0% (7/141)
Doxycycline	0.8% (1/127)	0.0% (0/14)	0.7% (1/141)
Chloramphenicol	0.0% (0/127)	14.3% (2/14)	1.4% (2/141)
Total koalas receiving one antibiotic	75.6% (96/127)	64.3% (9/14)	74.5% (105/141)
Amoxicillin (long-acting) and duplocillin	2.4% (3/127)	0.0% (0/14)	2.1% (3/141)
Amoxicillin (long-acting) and amoxicillin/clavulanic acid	8.7% (11/127)	0.0% (0/14)	7.8% (11/141)
Amoxicillin (long-acting) and ceftazidime	0.8% (1/127)	0.0% (0/14)	0.7% (1/141)
Amoxicillin (long-acting) and enrofloxacin	8.7% (11/127)	0.0% (0/14)	7.8% (11/141)
Amoxicillin/clavulanic acid and enrofloxacin	0.8% (1/127)	7.1% (1/14)	1.4% (2/141)
Amoxicillin/clavulanic acid and trimethoprim/sulfamethoxazole	0.8% (1/127)	0.0% (0/14)	0.7% (1/141)
Chloramphenicol and enrofloxacin	0.0% (0/127)	28.6% (4/14)	2.8% (4/141)
Total koalas receiving two antibiotics	22.0% (28/127)	35.7% (5/14)	23.4% (33/141)
Amoxicillin (long-acting), amoxicillin/clavulanic acid and enrofloxacin	2.4% (3/127)	0.0% (0/14)	2.1% (3/141)
Total koalas receiving three antibiotics	2.4% (3/127)	0.0% (0/14)	2.1% (3/141)

Supplementary Table 2. Outcome of 141 koalas which received antibiotic treatment during the 2019-2020 wildfires.

Koala outcome	Facility A	Facility B
Died in-care	34.6% (44/127)	35.7% (5/14)
Euthanised	29.1% (37/127)	14.3% (2/14)
Released	9.4% (12/127)	35.7% (5/14)
Transferred to another facility	0.0% (0/127)	7.1% (1/14)
Escaped enclosure (self-released)	3.1% (4/127)	0.0% (0/14)
Retained in captivity	0.0% (0/127)	7.1% (1/14)
Unknown	23.6% (30/127)	0.0% (0/14)
